# Relationship between cumulative exposure to occupational lifting throughout working life and risk of ischemic heart disease in men and women. The Copenhagen Aging and Midlife Biobank

**DOI:** 10.1093/annweh/wxad077

**Published:** 2023-12-24

**Authors:** Mette Korshøj, Anne Møller, Volkert Siersma, Rikke Lund, Charlotte Ø Hougaard, Ole S Mortensen, Karen Allesøe

**Affiliations:** Department of Occupational and Social Medicine, Holbæk Hospital, part of Copenhagen University Hospital, Gl. Ringstedvej 4B, 4300 Holbæk, Denmark; The Research Unit for General Practice and Section of General Practice, Department of Public Health, University of Copenhagen, Øster Farimagsgade 5, build. 24 Q, 1353 Copenhagen K, Denmark; The Research Unit for General Practice and Section of General Practice, Department of Public Health, University of Copenhagen, Øster Farimagsgade 5, build. 24 Q, 1353 Copenhagen K, Denmark; Section of Social Medicine, Department of Public Health, University of Copenhagen, Øster Farimagsgade 5, build. 24 Q, 1353 Copenhagen K, Denmark; Center for Healthy Aging, Faculty of Health Sciences, University of Copenhagen, Panum, building 7.2 & 7.3, Blegdamsvej 3B, 2200 Copenhagen N, Denmark; Section of Social Medicine, Department of Public Health, University of Copenhagen, Øster Farimagsgade 5, build. 24 Q, 1353 Copenhagen K, Denmark; Department of Occupational and Social Medicine, Holbæk Hospital, part of Copenhagen University Hospital, Gl. Ringstedvej 4B, 4300 Holbæk, Denmark; Section of Social Medicine, Department of Public Health, University of Copenhagen, Øster Farimagsgade 5, build. 24 Q, 1353 Copenhagen K, Denmark; Department of Occupational and Social Medicine, Holbæk Hospital, part of Copenhagen University Hospital, Gl. Ringstedvej 4B, 4300 Holbæk, Denmark; Center for Clinical Research and Prevention, Copenhagen University Hospital – Bispebjerg and Frederiksberg, Copenhagen, Hovedvejen, Entrance 5, Nordre Fasanvej 57, 2000 Frederiksberg, Denmark

**Keywords:** cardiovascular disease, cardiovascular risk, heavy lifting, IHD, occupational physical activity, occupational exposure, occupational medicine, prevention of cardiovascular disease

## Abstract

Ischemic heart disease (IHD) causes mortality and morbidity. High levels of occupational physical activity (OPA) increases IHD risk, and occupational lifting (OL) is suggested as a detrimental OPA exposure. This study investigated the association between accumulated OL throughout working life, and risk for IHD, and potential sex and hypertension differences. Data from Copenhagen Ageing and Midlife Biobank linked to register-based information on incident IHD during 9 years follow-up in the Danish National Patient Registry were included. The outcome was the odds of IHD from baseline (2009–2011) to end of follow-up (2018), among participants without IHD at baseline. Accumulated OL was assessed by linking occupational codes to a Job Exposure Matrix, creating a measure in ton-years (lifting 1,000 kg/day/year). Multivariable logistic regression tested associations between level of accumulated OL and IHD, among the 6,606 included individuals (68% men). During follow-up, 7.3% men and 3.6% women were hospitalized with IHD. Among all participants, the odds for IHD were 47% (OR 1.47, 95% CI 1.05–2.06) higher among those with ≥5 to <10 ton-years, 39% (OR 1.39, 95% CI 1.06–1.83) higher among those with ≥10 to <30 ton-years, and 62% (OR 1.62, 95% CI 1.18–2.22) higher among those with ≥30 ton-years, compared to no accumulated OL. However, these increased odds were in the same direction in the fully-adjusted model but statistically insignificant, ≥5 to <10 ton-years OR 1.28, 95% CI 0.88–1.88; ≥10 to <30 ton-years OR 1.20, 95% CI 0.85–1.69; and ≥30 ton-years OR 1.22, 95% CI 0.81–1.84. No statistically significant interactions, nor any associations, between OL and sex, or hypertension were seen.

**What’s important about this paper?**

High levels of occupational physical activity increases risk for ischemic heart disease. This study found the odds for ischemic heart disease were 47–62% higher among those with ≥5 ton-years of accumulated lifting, compared to no accumulated occupational lifting, though the increased odds were not statistically significant in the fully-adjusted model. This study suggests that occupational lifting may be a detrimental occupational physical activity.

## Introduction

Cardiovascular disease (CVD) is the leading cause of death and disease burden both globally and in Europe (Vaduganathan et al. 2022). Ischemic heart disease (IHD) is the main CVD, accounting for 9.44 million deaths in 2021 (Vaduganathan et al. 2022). There is solid evidence that being physically active during leisure time (LTPA) promotes health and decreases the risk of many chronic diseases including CVDs (Warburton et al. 2006; Löllgen and Papadopoulou 2018). In contrast, a high level of occupational physical activity (OPA) has been shown to associate to a higher risk of CVD and all-cause mortality (Li et al. 2013; Coenen et al. 2018; Holtermann et al. 2021). One of the suggested differences behind this seemingly opposing effect on cardiovascular health, from OPA and LTPA, is the higher amount of static versus dynamic physical activity, the duration of this exposure, and the possibility of recovering between strenuous tasks (Vanhees et al. 2012; Holtermann et al. 2018). Occupational lifting (OL) is a potentially detrimental form of static physical activity. Lifting may impose strain on the cardiovascular system through elevated blood pressure and heart rate both acutely and after work (MacDougall et al. 1985; Clays et al. 2012), both factors known to increase the risk of heart disease (Archangelidi et al. 2018; Hermansen et al. 2019). Even with low force demands, static physical activity, such as lifting, can be strenuous, if it is of long duration or high frequency.

The labor market is highly gender segregated as there are typical male and female occupations, especially in jobs with high OPA (Aittomäki et al. 2005). Also, within the same occupational title are differences across exposure to OPA seen to be gender segregated (Messing et al. 1998; Eng et al. 2011;Smith et al. 2018). Women in jobs with high OPA and OL typically work in the health care, social care, kitchen, or cleaning sectors, whereas men exposed to OL held a variety of jobs (Aittomäki et al. 2005; Møller et al. 2013). Besides these sex differences in exposure to OPA and OL, there may also exist sex differences in the physiological effects from OPA and OL, based on differences in basal metabolism, physical capacity, muscle mass, strength, and sex hormones between men and women (Ilmarinen 1989; Søgaard and Sjøgaard 2015). These physiological differences in effects from OPA and OL might contribute to the explanation of the average 7–10 years later first manifestation of heart diseases in women compared to men (Maas and Appelman 2010). Therefore, examining sex differences of the health effects of OPA and OL is warranted in occupational health research (Guettler 2023; Coenen et al. 2020).

Some previous studies have reported on associations between OL and risk for CVDs. Petersen and colleagues (Petersen et al. 2012) found contrasting associations between heavy OL and risk of IHD among men and women, where men showed higher risks, but not women. On the other hand, similar associations have been suggested between both repetitive and heavy OL, and increased risk of myocardial infarction (MI) among both men and women (Fransson et al. 2004). In contrast, a study from Norway does show a significantly lower risk of cardiovascular mortality among both men and women exposed to a combination of walking and OL, compared to those mainly sedentary at work (Hermansen et al. 2019). In a Danish study among men, based on expert valuation of exposure to OL in different occupations, no association between working in occupations involving lifting and risk of IHD was identified (Hannerz and Holtermann 2016). Thus, the results are contrasting and further investigations are needed to clarify whether OL is a risk factor for CVD and whether the associations differ across sex.

Although technical solutions have been made to handle the most strenuous tasks, more than 35% of European workers in 2021 stated to be carrying or handling heavy loads at work (European working conditions survey 2021), 41% of men and 29% of women. Additionally, 15% of women, and only 4% of men working in Europe stated exposure to moving and lifting of people at work (European working conditions survey 2021).

Moreover, previous studies have indicated increased vulnerability to high OPA exposure among workers with existing CVDs (Allesøe et al. 2016; Korshøj *et al.* 2023; Krause 2010). And, along the same lines, OL, where the blood pressure is elevated, are proposed as being especially detrimental to workers with hypertension (Korshøj *et al.* 2020; Williamson et al. 2016).

Thus, the present study has the following 3 aims: (i) to investigate the association between accumulated OL throughout working life, and risk for IHD measured as 9-year incidence after participation in the Copenhagen Aging and Midlife Biobank (CAMB) (Lund et al. 2016) examination, in late working life; (ii) to investigate potential sex differences in this association; and (iii) to investigate whether workers medicated for hypertension from 1995 to baseline are at higher risk for developing IHD than normotensive workers when exposed to OL. We hypothesize that heavy OL accumulated through many years is associated with an increased risk of IHD, both among men and women, and that workers with hypertension are at increased risk of developing IHD from OL.

## Methods

### Study design, study population, exclusion criteria, and baseline

This prospective cohort study is based on data from CAMB with individual linkage to register-based information on incident IHD during 9 years follow-up in the Danish National Patient Registry. CAMB consists of participants from 3 cohort studies: The Copenhagen Perinatal Cohort (Merrick et al. 1983), the Danish Longitudinal Study on Work, Unemployment and Health (Christensen et al. 2004), and the Metropolit Cohort (Osler et al. 2006). In the CAMB study, 17,939 men and women, aged 49–63 years, were invited to participate by questionnaire and clinical examination in 2009 to 2011. Hereof, 7,191 returned the self-administered questionnaire on sociodemographic and socioeconomic factors, health and medication, lifestyle, and working conditions. The baseline for each participant was the first date of clinical examinations for those only answering a questionnaire and the exact date of clinical examination for those both participating both with a questionnaire and in the clinical examination.

In the present study, the study population consisted of 6,606 individuals, hereof 4,510 men and 2,096 women, who answered the questionnaire, had information about accumulated OL, and with no record of hospital admission from IHD prior to baseline.

### Outcomes

The outcome was the risk of incident IHD from baseline in 2009–2011 to end of follow-up in 2018, among participants free from IHD at baseline. Additionally, in a secondary analysis, risk of mortality during follow-up was used as outcome. Information about incident IHD was retrieved from the Danish National Patient Registry (Schmidt et al. 2015) by linkage of the personal identification number allocated to all residents in Denmark. This register was also consulted to exclude study participants with occurrences of IHD prior to baseline from the study population. IHD was defined according to the WHO International Classification of Diseases (ICD 10 (from 1994) and ICD 8 (from 1977 until the end of 1993)) as hospitalization for myocardial infarction (410 in ICD-8 and I21-23 in ICD-10), other acute or chronic IHD (411-412 in ICD-8 and I24-25 in ICD-10), angina (413 in ICD-8 and I20 in ICD-10), or electrocardiographically diagnosed heart disease (414 in ICD-10). Information about mortality during follow-up was retrieved from the Danish Registry of Causes of Deaths. Information on date of death was retrieved from the Central Person Registry.

### Assessment of accumulated occupational lifting

Accumulated OL was assessed by a linkage of occupational codes from reported job history to a Job Exposure Matrix (JEM), whereby a continuous measure in ton-years (lifting 1,000 kg/day/year) was formed. Information about job history: job title, type of workplace, and employment period (in calendar-year) in the 5 longest held positions, during work-life, and the present position was self-reported in the CAMB questionnaire. Based on this job history, occupational codes according to the Danish version of the International Standard Classification of Occupations register (D-ISCO-88), were assigned to each separate employment (Møller et al. 2012). The JEM used in this study was The Lower Body JEM (Rubak et al. 2014). The JEM was originally developed for investigating the association between physical work demands and risk of osteoarthritis in lower limbs. Levels of exposure to lifting (amount of daily lifting per day and number of heavy lifts of more than 20 kg), sitting, standing/walking, whole body vibration, and kneeling was assigned to each occupational code by experts (Møller et al. 2012). For each participant, ton-years were calculated for each employment, by multiplying years in this employment, by corresponding amount of lifting per day, retrieved from linkage of the occupational code to the JEM. Ton-years was thereafter calculated to give a standardized measure of exposure to lifting during the entire working life (Møller et al. 2012). A categorical variable was also formed, based on the distribution of exposure to OL in the included population, (i) No exposure (reference group), (ii) low exposure (>0–5 ton-years), (iii) moderate exposure (>5 to <10 ton-years), (iv) high exposure (≥10 to <30 ton-years), and (v) very high exposure ≥30 ton-years.

### Assessment of covariates

From available covariates, potential confounders for the association between accumulated OL and IHD were selected a priori, based on current knowledge from the literature (Gielen et al. 2015; Kivimäki and Kawachi 2015). Information about sex and age at baseline was retrieved from the Central Person Registry. A categorical age variable was included in the descriptive analyses: 49–54; 55–59; 60–63. In all other analyses, age was included as a continuous variable.

Cohabitation status was assessed by 2 questions: “Do you live alone” (yes/no) and “Do you live with – spouse/partner” (yes/no) and categorized as (i) Living alone; or (ii) Married/cohabitating.

Vocational education was assessed in the questionnaire and categorized as (i) unskilled; (ii) skilled; (iii) short; (iv) medium; or (v) long higher education.

Self-rated fitness was assessed by the question: How do you rate your fitness level compared to peers (same sex and age) in 9 categories, where 5 is the same level as peers. It was categorized as (i) low = 1–3; (ii) medium = 4–6; or (iii) high = 7–9.

Self-rated muscular strength was assessed by a question: How do you rate your muscular strength compared to peers (same sex and age) in 9 categories, where 5 is the same level as peers. It was categorized as (i) low = 1–3; (ii) medium = 4–6; or (iii) high = 7–9.

Body mass index (BMI) was a continuous measure calculated from self-reported height and weight as weight in kilograms divided by square of height in meters.

Assessed by questionnaire: “Are you suffering from, or have you previously suffered from diabetes” (yes/no), or ever use of ATC code A10, covering all kinds of diabetes medication, based on information from The Danish National Prescription Registry from 1995, when the registry was initiated until baseline.

A high level of cholesterol was assessed as use of lipid-lowering medication: Information from The Danish National Prescription Registry about ever having used lipid-lowering medication (ATC code C10) from 1995 until baseline.

Smoking was assessed by questionnaire by a single item: “Do you smoke?” and categorized as (i) current smoker (daily and not daily) or (ii) former smoker or never smoker.

Continuous variable of average intake of beer, wine, and spirits per week calculated in units (10 ml or 8 g of pure alcohol).

Psychosocial work factors were assessed by a modified version of Copenhagen Psychosocial Questionnaire (COPSOQ) (Kristensen and Borg 2003) by 12 single questionnaire items; of these, 5 items were selected for this analysis. Four of the items (quantitative demands, work pace, cognitive demands, and emotional demands) constituted “demands at work” (Cronbach’s α = 0.57). Influence at work was assessed by a single item: “do you have a lot of influence on the decisions about your work?,” categorized as (i) always or often; (ii) sometimes; and (iii) rarely or never/almost never.

Hypertension was assessed by self-report yes/no or ever use of ATC code: C02-04, C07-09, covering all kinds of anti-hypertensive medication, from The Danish National Prescription Registry between 1995 and baseline.

### Statistical methods

The distribution of the level of exposure variables and covariates in the study population is reported as percentages for categorical variables, or as means and standard deviations (SD), for continuous variables and reported for men and women, and according to level of lifting in 5 categories.

Odds ratios (OR) and 95% confidence intervals (CI) from multivariable logistic regressions were used to assess the associations between level of accumulated OL and incident IHD and mortality, respectively. It was decided a priori to investigate sex differences in the associations and two sets of analyses were performed: (i) in models including both men and women further adjusted for sex and (ii) in models including an interaction term between OL and sex, from which separate associations for men and women were derived.

Model 1 was adjusted for age in both A and B. Model 1a additionally adjusted for sex. For both A and B, model 2 included additional adjustment for a set of lifestyle factors and risk factors for IHD (smoking, alcohol, BMI, diabetes, and use of lipid-lowering medication); model 3 was adjusted for self-rated muscular strength and fitness; model 4 was adjusted for vocational education, civil status, and psychosocial occupational factors: demands at work and influence at work; and model 5 was the fully-adjusted model including all above-listed covariates.

In a sensitivity analysis, the association between OL and IHD was investigated in the age- and sex-adjusted model 1a among workers with and without hypertension. Interaction between accumulated OL and hypertension was tested by including an interaction term.

In the logistic regression analysis, the length of the follow-up time of each individual is not accounted for, and a number of participants die or can no longer be followed in the registries due to emigration, these participants therefore have an artificially lower risk of experiencing IHD in the logistic regression analysis. Therefore, in a sensitivity analysis, the association between accumulated OL and all-cause mortality was examined. Yet, in another sensitivity analysis, the association between accumulated OL and risk of IHD or mortality was examined to investigate the case in where all deaths hypothetically were caused by IHD.

In all analyses, *P* < 0.05 was considered statistically significant. Statistical analyses were performed using the statistical package SAS version 9.4.

## Results

The study population consisted of 6,606 respondents, hereof 4,510 men and 2,096 women. During follow-up, 327 (7.3%) men and 76 (3.6%) women were admitted to hospital with IHD, 206 (4.6%) men and 57 (2.7%) women died, and 28 (0.6%) men and 9 (0.4%) women emigrated or could no longer be followed in the registers. There were 2,030 men and 4,276 women, who were followed until the end of the follow-up period. Table 1 presents the baseline characteristics of the study population among men and women. The mean age was 55.1 years among men and 52.6 years among women. Table 2 represents the number of participants according to accumulated OL and cases of IHD.

**Table 1.** Characteristics (number and percentages) among 4,510 men and 2,096 women participating in the Copenhagen Ageing and Midlife Biobank, stratified on sex.

		All participants, *n* = 6,606			Men, *n* = 4,510			Women, *n* = 2,096		
		n	%	Mean (SD)	n	%	Mean (SD)	n	%	Mean (SD)
**Age**	**Mean age (years)**	6,606		54.3 (4.0)	4,510		55.1 (3.4)	2,096		52.6 (4.5)
**Civil status**	**Married/cohabitating**	5,108	79.4		3,566	80.9		1,542	76.0	
	**Living alone**	1,329	20.6		842	19.1		487	24.0	
	**Missing**	169								
**Vocational education**	**None/semiskilled**	785	12.0		562	12.6		223	10.7	
	**Skilled**	2,373	36.2		1,663	37.2		710	34.2	
	**Short/middle longer**	2,211	33.7		1,358	30.3		853	41.1	
	**Long academic**	1,126	17.2		850	19		276	13.3	
	**Missing**	54								
**Ton-years - continuously**				10.4 (20.0)			12.5 (12.5)			5.9 (1.8)
**Ton-years**	**0**	3,066	46.4		1,937	43.0		1,129	53.9	
	**>0 and <5**	967	14.6		629	14.0		338	16.1	
	**≥5 and <10**	664	10.1		460	10.2		204	9.7	
	**≥10 and <30**	1,247	18.9		896	19.8		351	16.8	
	**≥30**	666	10.0		588	13.0		74	3.5	
**Leisure time physical activity**	**Vigorous**	155	2.4		127	2.8		28	1.4	
	**Moderate**	1,902	29.0		1,393	31.1		509	24.4	
	**Light**	3,825	58.4		2,453	54.8		1,372	66.0	
	**Sedentary**	671	10.2		500	11.2		171	8.2	
	**Missing**	53			-			-		
**Smoking**	**Current**	1,687	25.6		1,169	26.0		518	24.8	
	**Never/former**	4,899	74.2		3,326	74.0		1,573	75.2	
	**Missing**	20			-			-		
**Alcohol intake**	**Intake Units/week**	6,499		12.3 (13.1)			14.7 (14.4)			7.1 (7.3)
	**Missing**	107								
**Self-rated fitness**	**Better than peers**	2,350	35.8		1,779	39.7		571	27.3	
	**Same as peers**	3,153	48.0		2,093	46.7		1,060	50.8	
	**Worse than peers**	1,066	16.2		610	13.6		456	21.9	
	**Missing**	20								
**Self-rated muscular strength**	**Better than peers**	2,387	36.3		1,834	40.9		553	26.5	
	**Same as peers**	3,543	53.9		2,362	52.7		1,181	56.6	
	**Worse than peers**	638	9.7		285	6.4		353	16.9	
	**Missing**	38								
**BMI continuous kg/m** ^**2**^		6,507		26. (6.1)			26.6 (5.0)			25.5 (8.0)
	**Missing**	1,420								
**Diabetes (self-reported)**	**No**	6,116	92.6		4,129	91.4		1,996	95.2	
	**Yes**	490	7.4		390	8.6		100	4.8	
	**Missing**									
**Use of lipid-lowering medication**	**No**	4,820	73.0		3,157	70.0		1,663	79.3	
	**Yes**	1,786	27.0		1,353	30.0		433	20.7	
**Work demand scale**				3.07 (0.67)			3.08 (0.66)			3.06 (0.68)
**Work influence**	**Always/often**	4,782	72.8		3,432	76.5		1,350	64.8	
	**Sometimes**	1,264	19.2		742	16.5		522	25.1	
	**Seldom/never/hardly**	523	8.0		312	7.0		211	10.1	
	**Missing**	37								
**Use of anti-hypertensive medication**	**No**	3,737	56.6		2,473	54.8		1,264	60.3	
	**Yes**	2,869	43.4		2,037	45.2		832	39.7	
**Self-reported hypertension**	**No**	4,931	75.2		3,277	73.3		1,654	79.3	
	**Yes**	1,629	24.8		1,196	26.7		433	20.7	
	**Missing**	46								
**Hypertension** ^**#**^	**No**	3,514	53.4		2,304	51.3		1,210	57.8	
	**Yes**	3,068	46.6		2,185	48.7		883	42.2	
	**Missing**	24								
**Menopause**	**No**							868	41.9	
	**Yes**							1,206	58.1	
	**Missing**							22		

**Table 2.** Number of participants and number of cases of IHD according to level of accumulated occupational lifting among 4,510 men and 2,096 women participating in the Copenhagen Ageing and Midlife Biobank.

Ton-years of occupational lifting	All participants			Men			Women		
	*n*	n IHD	% IHD	*n*	n IHD	% IHD	*n*	n IHD	% IHD
**0**	3,066	160	5.2%	1,816	121	6.7%	1,090	39	3.6%
**>0 and <5**	967	52	5.4%	591	38	6.4%	324	14	4.3%
**≥5 and <10**	664	47	7.1%	421	39	9.3%	196	8	4.1%
**≥10 and <30**	1,250	144^*^	7.8%	822	129^*^	9.5%	338	15^*^	3.7%
**≥30**	605			533			72		

^*^Due to anonymization, n IHD is the sum of ≥10 and <30 and ≥30.

Table 3 presents the association between accumulated OL in ton-years in 5 categories and IHD among all participants, and men and women separately. Among all participants, the age-adjusted OR for IHD was 47% higher among those with ≥5 and <10 ton-years, 39% higher among those with accumulated OL of ≥10 and <30, and 62% higher among those with ≥30 ton-years compared to those with no accumulated OL (Table 3). Those with >0 and <5 ton-years had a small but statistically insignificant increased risk of IHD. Adjustment for sex in model 1a attenuated the estimates, especially among those with the highest level of lifting, but all estimates remained statistically significant in model 1. However, adjustment for a set of lifestyle factors and risk factors for IHD (smoking, alcohol, BMI, diabetes, and use of lipid-lowering medication) in model 2, adjustment for muscular strength, and self-rated fitness in model 3, or adjustment for vocational education, civil status, and psychosocial occupational factors in model 4, attenuated the estimates and none of them were statistically significant. In the final model 5, there was a tendency of an increased risk of IHD from OL of 5 or above ton-years, but none of these estimates were statistically significant.

**Table 3.** Odds ratio (OR) for IHD according to level of accumulated occupational lifting among 6,606 individuals, and 4,510 men and 2,096 women participating in the Copenhagen Ageing and Midlife Biobank (CAMB) 2009-11. 403 cases of IHD, hereof 327 among men and 76 among women during follow-up.

Accumulated occupational lifting (ton-years)	All		Men		Women	
	OR	95% CI	OR	95 % CI	OR	95 % CI
^a^ **Model 1**						
0	1	-	1	-	1	-
>0 and <5	1.11	0.81–1.54	1.02	0.70 – 1.49	1.33	0.71 – 2.49
≥5 and <10	**1.47**	**1.05–2.06**	**1.46**	**1.00 – 2.13**	1.26	0.58 – 2.74
≥10 and <30	**1.39**	**1.06–1.83**	**1.39**	**1.03 – 1.87**	1.13	0.60 – 2.14
≥30	**1.62**	**1.18–2.22**	**1.54**	**1.11 – 2.15**	0.73	0.17 – 2.98
^b^ **Model 1a**						
0	1	-				
>0 and <5	1.09	0.79–1.50				
≥5 and <10	**1.41**	**1.01–1.99**				
≥10 and <30	**1.33**	**1.02–1.75**				
≥30	**1.47**	**1.07–2.02**				
^c^ **Model 2**						
0	1	-	1	-	1	-
>0 and <5	1.01	0.73**–**1.41	0.96	0.65 - 1.40	1.22	0.64 - 2.32
≥5 and <10	1.33	0.95**–**1.87	1.36	0.93 - 1.99	1.24	0.57 - 2.70
≥10 and <30	1.21	0.92**–**1.59	1.24	0.91 - 1.69	1.08	0.57 - 2.05
≥30	1.33	0.96**–**1.84	1.38	0.98 - 1.94	0.74	0.17 - 3.14
^d^ **Model 3**						
0	1	-	1	-	1	-
>0 and <5	1.02	0.74**–**1.42	0.97	0.66 – 1.42	1.23	0.64 – 2.34
≥5 and <10	1.29	0.91**–**1.82	1.30	0.89 – 1.92	1.23	0.56 – 2.68
≥10 and <30	1.19	0.90**–**1.57	1.22	0.90 – 1.66	1.06	0.56 – 2.01
≥30	1.30	0.94**–**1.81	1.35	0.96 – 1.90	0.74	0.17 – 3.13
^e^ **Model 4**						
0	1	-	1	-	1	-
>0 and <5	0.99	0.71**–**1.40	0.93	0.62 – 1.37	1.26	0.65 – 2.48
≥5 and <10	1.38	0.97**–**1.97	1.37	0.93 – 2.04	1.43	0.65 – 3.15
≥10 and <30	1.15	0.84**–**1.58	1.18	0.83 – 1.67	1.03	0.51 – 2.08
≥30	1.24	0.86**–**1.80	1.29	0.87 – 1.90	0.71	0.16 – 3.03
^f^ **Model 5**						
0	1	-	1	-	1	-
>0 and <5	0.94	0.66**–**1.36	0.88	0.58 - 1.34	1.22	0.59 - 2.50
≥5 and <10	1.28	0.88**–**1.88	1.26	0.82 - 1.92	1.41	0.59 - 3.37
≥10 and <30	1.20	0.85**–**1.69	1.24	0.85 - 1.80	0.95	0.45 - 1.99
≥30	1.22	0.81**–**1.84	1.25	0.81 - 1.91	0.71	0.16 - 3.16

^a^Model 1: Adjusted for age. ^b^Model 1a: Adjusted for age and sex. ^c^Model 2: Adjusted for age, sex, diabetes, BMI, smoking and alcohol consumption, and use of lipid-lowering medication. ^d^Model 3: Adjusted for age, sex, self-rated fitness, and self-rated muscle strength. ^e^Model 4: Adjusted for age, sex, vocational education, civil status, and psychosocial work factors: work demands and influence at work. ^f^Model 5: Full models including all covariates.

BMI, body mass index; CI, confidence interval; IHD, ischemic heart disease; OR, odds ratio.

Among men and women, there was no statistically significant interaction between OL and sex (*P* = 0.670 in the age-adjusted and *P* = 0.979 in the fully-adjusted model 5). Among men, a statistically significant association between level of OL and IHD was seen in the age-adjusted analysis, as OR for IHD was 46% higher among those with ≥5 and <10 ton-years, 39% higher among those with accumulated OL of ≥10 and <30, and 54% higher among those with ≥30 ton-years compared to those with no accumulated OL (Table 3).

Adjustment in models 2–4 attenuated the estimates and the associations were no longer statistically significant. In the fully-adjusted model 5, a modest increased risk of IHD among those with a level of OL of ≥5 ton-years, was still indicated, but this was not statistically significant.

Among women, an increased risk of IHD was indicated, but not statistically significant, among those with >0 to <5 and ≥5 and <10 ton-years, and a lower risk of IHD was indicated among those with ≥30 ton-years, compared to those without OL in models 1, 2, 3, and 4. In the fully-adjusted model, the same pattern was seen but none of the estimates were statistically significant.

A sensitivity analysis adjusted for age, sex, vocational education, and civil status was performed as some of the confounders might be considered as mediators rather than confounders. This sensitivity analysis did not show numerically different OR than reported in model 1a and 1 (Table 3). Moreover, this sensitivity analysis showed solely non-significant associations.

Table 4 presents the association between the level of accumulated OL in ton-years in 5 categories and IHD among all participants, stratified by whether the participants were hypertensive or normotensive. Among hypertensive workers, there was a tendency of higher risk of IHD among all groups with OL, ranging from 14% higher among those with >0 and <5 ton-years to 36%, among those with ≥30 ton-years, compared to those with no accumulated OL in the age-adjusted analysis (Table 4). This association was, however, not statistically significant and the estimates were attenuated in the fully-adjusted model. In contrast, high accumulated OL did not seem to be associated with IHD among normotensive workers. Normotensive workers exposed to lifting of >0 and <5 ton-years, actually seemed to have a lower risk of IHD. This association was, however, not statistically significant. There was no statistically significant interaction between OL and being hypertensive or normotensive (*P* = 0.776 in the fully-adjusted model), and we cannot rule out that the observed differences are chance findings.

**Table 4.** Odds ratio (OR) for IHD according to level of accumulated occupational lifting and being normotensive or hypertensive among 4,510 men and 2,096 women, participating in the Copenhagen Ageing and Midlife Biobank (CAMB) 2009-11. 403 cases of IHD hereof 327 among men and 76 among women during follow-up.

Accumulated occupational lifting (ton-years)	Normotensive		Hypertensive	
	OR	95 % CI	OR	95 % CI
^a^ **Model 1**				
0	1	-	1	-
>0 and <5	0.56	0.22–1.45	1.14	0.80–1.63
≥5 and <10	1.04	0.43–2.52	1.33	0.91–1.94
≥10 and <30	0.97	0.48–1.95	1.29	0.95–1.75
≥30	0.98	0.41–2.39	1.36	0.95–1.92
				
^**b**^ **Model 5**				
0	1	-	1	-
>0 and <5	0.56	0.21–1.46	1.06	0.71–1.59
≥5 and <10	1.08	0.44–2.68	1.28	0.84–1.96
≥10 and <30	0.92	0.43–1.96	1.26	0.87–1.83
≥30	1.01	0.40–2.54	1.24	0.80–1.92

^a^Model 1: Adjusted for age. ^b^Model 5: Adjusted for age, diabetes, BMI, smoking and alcohol consumption and use of lipid-lowering medication, self-rated fitness and self-rated muscle strength, vocational education, civil status, and psychosocial work factors: work demands and influence at work.

*P*-value accumulated occupational lifting × hypertension: Model 1: *P* = 0.692; model 5: *P* = 0.776.

BMI, body mass index; CI, confidence interval; IHD, ischemic heart disease; OR, odds ratio.

A sensitivity analysis among all participants of the association between OL and all-cause mortality, adjusted for age and sex, showed a similar pattern as with IHD as outcome: Compared to those without OL, OR for all-cause mortality was 0.97 (95% CI 0.64–1.48) among those with ton-years >0 and <5, 1.30 (95% CI 0.84–2.01) among those with ton-years ≥5 and <10, 1.52 (95% CI 1.10–2.11) among those with ton-years ≥10 and <30, and 1.98 (95% CI 1.38–2.86) among those with ≥30 ton-years.

Yet, a sensitivity analysis of the association between OL and IHD and all-cause mortality, as outcome, was performed to see the result if all deaths during follow-up could be attributed to IHD. This analysis among all participants, adjusted for age and sex, showed a similar pattern as with IHD as outcome: Compared to those without OL, OR for all-cause mortality was 1.06 (95% CI 0.81–1.38) among those with ton-years >0 and <5, 1.38 (95% CI 1.05–1.84) among those with ton-years ≥5 and <10, 1.36 (95% CI 1.09–1.70) among those with ton-years ≥10, and <30 and 1.73 (95% CI 1.34–2.23) among those with ≥30 ton-years.

Additionally, adjusting for menopause among the women did not affect the presented associations among women.

## Discussion

### Main results

In the age- and sex-adjusted analyses, among all participants and men separately, stated that the higher the accumulated lifting burden, the higher odds of IHD. Specifically, 47% higher among those with ≥5 and <10 ton-years, 39% higher among those with accumulated OL of ≥10 and <30, and 62% higher among those with ≥30 ton-years, compared to those with no accumulated OL. However, adjustment for IHD risk factors, leisure time physical activity, civil status, vocational education, and psychosocial work factors, all attenuated the estimates and none of the estimates remained statistically significant. A number of other factors, associated with increased risk of IHD, therefore, seems to explain the associations seen in the age- and sex-adjusted models.

We also investigated if the association between accumulated OL and IHD differed between men and women. The interaction analysis showed no statistically significant interaction between OL and sex, as well as between OL and hypertension status; therefore, the differences in the associations among men and women might be chance findings.

### Previous knowledge

The association between OL and risk of IHD has been sparsely investigated and with mixed results (Fransson et al. 2004; Hannerz and Holtermann 2016; Petersen et al. 2012). Hannerz and Holtermann find no associations between men employed in occupational groups exposed to heavy lifting and risk of IHD (Hannerz and Holtermann 2016), which corresponds to the findings from this study among men. Petersen and colleagues (Petersen et al. 2012) find no associations between OL and risk for IHD among women, but a significantly increased risk among men in a model adjusted for lifestyle, LTPA, OPA, and education. Thus, these results (Petersen et al. 2012) mimic the results in this paper in the model only adjusting for age. Lastly, Fransson and colleagues (Fransson et al. 2004) report a lower risk (22%) for low HDL cholesterol among male workers stating to be exposed to heavy OL, in an analysis adjusted for age, smoking, LTPA, and socioeconomic status. As low HDL cholesterol is a risk factor for IHD (Sniderman et al. 2011), this could be interpreted as exposure to heavy OL might lower risk for IHD among men, which corresponds to our finding of no association among men in the fully-adjusted model. In summary, the sparse previous knowledge on the association between OL and IHD is inconclusive, and the results from the present study do not contribute to a clearer understanding of the association. Rather one could speculate whether the association between OL and risk for IHD is explained by socioeconomics as adjustment for covariates related to socioeconomic status showed associations in similar direction, but insignificant. However, hypothetically OL would lead to increases in the blood pressure, as every lift acutely increases the total peripheral resistance due to the occlusion of vessels during the static muscle activity (MacDougall et al. 1985; Saladini et al. 2016), and a raised blood pressure or hypertension are stated as one of the main risks for IHD across both sexes (Bergami et al. 2022). In the literature, the results are mixed on the association between OL and blood pressure (Baumann et al. 2023; Clays et al. 2012; Korshøj *et al.* 2020; Korshøj *et al.* 2021), leading to speculations of other pathways for explanation of the increased risk for CVD among workers with high OPA (Holtermann *et al.* 2021.), accuracy in the estimation of OL (Korshøj *et al.* 2022b) or impacts by the healthy worker selection bias (Li and Sung 1999). Nevertheless, none of the previous studies, have included an accumulated estimate of the OL, but have been based on exposure to OL at one time-point (Fransson et al. 2004; Petersen et al. 2012; Hannerz and Holtermann 2016). Although this study includes an accumulated estimate of OL, the risk of healthy worker selection bias (Li and Sung 1999) could still affect the workers to migrate to less strenuous occupations, e.g. caused by the acute peaks in blood pressure which might give angina (Robinson 1967). Furthermore, future studies should consider to investigate the potential mediating, moderating, or confounding effect of OPA on the association between OL and IHD, as OL in most occupations is performed in combination with moderate to high levels of OPA (European working conditions survey 2021).

### Secondary analyses

The secondary aims of the study were to investigate potential differences in this association between OL and risk for IHD across sexes and hypertensive status. No interaction was seen between OL and sex, neither did the risk for IHD by exposure to OL seem to differ across sex, except in model 1, where the associations among men were statistically significantly showing increased risk for IHD by exposure to occupational lifting, and no associations were shown among women. Also, no interaction was seen between OL and hypertensive status, and the analysis stratified by hypertension did not show any differences in risk for IHD by exposure to OL.

To the best of our knowledge, no other studies have investigated the association between OL and IHD including an exploration of the potential effect modification by sex, by testing for interaction. Only a few studies investigating the association between OPA and IHD have tested for interaction, with mixed results (Holtermann 2021; Hu et al. 2014; Holtermann et al. 2016; Allesøe et al. 2023). One challenge in the examination of the association between OL and IHD among working women are the need for a large sample size as IHD is less frequent among working women than working men (Maas and Appelman 2010). Also, the gender segregation in the labor market and in the distribution of the physical workloads including OL (Eng et al. 2011; Møller *et al.* 2013) may impact this investigated association. Moreover, it is expected that the physiological sex differences of, e.g. physical capacity, as muscle mass and cardiorespiratory fitness, and sex hormones, as well as individual factors like occurrence of musculoskeletal pain or other trauma, affect both the level of relative intensity of the performed work task as well as the perception of strain (Ilmarinen 1989; Søgaard and Sjøgaard 2015).

### Strengths and limitations

The strengths of the present study are the register-based outcome of hospitalization due to IHD, being free of the biases related to self-reports and observations. Another strength is the choice of logistic regression, as it is (i) being robust to differences in distribution of the outcome across sub-groups, like men and women; (ii) allows for comparison across sub-groups, like men and women, within the same analysis; and (iii) produces risk estimates in OR, not being dependent on the baseline incidence of the outcome. Also, the unique feature of the accumulated exposure to OL is a strength, as the time period for the exposure reflects the majority of the work life for the participants. Nevertheless, this type of exposure assessment might also hold limitations as (i) the exposure were cumulated and then categorized; therefore, we cannot rule out that the same exposure category of OL might hold participants both exposed to high loads of OL for few years and low loads of OL for many years; (ii) having the same categories of OL for both sexes might blur the investigation, as men and women are exposed to OPA differently within the same occupational group (Eng et al. 2011); (iii) it does not account for the development in occupational exposures across a work life, e.g. due to changes in legislation, use of assistive devices, and changes in the tasks in the occupational titles due to ex digitalization and technological development; (iii) it does not account for individual adjustments in the performance of the OL, e.g. due to musculoskeletal disorders or other symptoms from, e.g. CVDs. Therefore, future studies could consider to combine this type of exposure with occupational titles to better investigate sex differences. Moreover, those with the highest loads of exposure to OL, and no IHD at baseline, could be a selected group, being especially fit for this type of exposure, as those not, would be expected to leave the occupation, this phenomena are called the healthy worker selection bias (Li and Sung 1999). This might draw the associations under investigation towards a null-finding. Regarding the adjustment, the significant OR, in Table 3, might turn insignificant due to over-adjustment as there are several overlapping pathways of IHD risks that are combined in this analysis, e.g. there can be an association between socioeconomic status and exposure to OL, as the majority of those having high OPA also have short or no education (Holtermann et al. 2021). Moreover, will the combination of static muscle contraction and external loads, during lifting, lead to an acute occlusion of the arteries, affecting the total peripheral resistance (MacDougall et al. 1985) and blood pressure (McArdle et al. 2010) to rise. These acute effects combined with other risk factors for IHD leading to increased total peripheral resistance, e.g. smoking and age, developing over time may not necessarily be accounted for by adjusting for baseline values. Moreover, future investigations of this association between OL and IHD risk could consider adjustment for family history of CVDs, as this might impact vulnerability to exposure to OL in relation to IHD risk. Furthermore, the null findings among women may be explained by the fact that the majority of women develop IHD at a higher age than men (Maas and Appelman 2010) and thus not in their working age, but when they are retired. In addition, these results should not be generalized to the general workforce, as this study population is in the end of their work life, thus these results are not generalizable to younger workers.

### Perspectives for prevention and future research

The results reported in this paper do not point towards prevention of IHD, among workers, being accomplished by reducing exposure to OL. However, association analysis between OL and IHD, stratified by sex, might still be warranted, and especially in non-Danish cohorts, as this is the only study investigating this besides the other Danish study by Petersen and colleagues (Petersen et al. 2012). However, overall prevention of CVD risk, among workers, could be accomplished by reducing exposure to OL, as OL (i) increase blood pressure, in some analysis (Clays et al. 2012; Korshøj *et al.* 2020), but not all (Korshøj *et al.* 2021); (ii) reduce function of the heart (Korshøj *et al.* 2022a; Nde *et al.* 2014); (iii) acutely increase the relative aerobic workload (Baumann et al. 2023); and (iv) lead to increased levels of inflammation (Feinberg et al. 2022). All of these are being factors leading to an increase in CVD risk (Lewington et al. 2002; Krause 2010; Pedersen et al. 2020), and are parts of the mechanism in the development of IHD, thus, future studies could consider to investigate this in mediation analysis. Also, future studies might also consider to investigate the combined effects of joined exposures of OPA and OL, as the literature shows high levels of OPA to increase risk for CVD (Coenen *et al.* 2018; Holtermann et al. 2021), however, without considering whether or not the OPA includes heavy OL.

## Conclusion

The age-adjusted analysis showed an increased risk for IHD among all participants and men, where accumulated exposure to OL ≥ 5 ton-years increased risk for IHD by 33–62%. However, the fully-adjusted analysis showed similar associations, although they were insignificant. No associations between accumulated OL and risk for IHD were seen in the analysis stratified by sex and hypertension.

## Data Availability

The data presented in this study are available on request from the CAMB steering committee. The data are not publicly available due to data protection regulations for individual level data.
